# VOC Characterization of *Byasa hedistus* (Lepidoptera: Papilionidae) and Its Visual and Olfactory Responses during Foraging and Courtship

**DOI:** 10.3390/insects15070548

**Published:** 2024-07-19

**Authors:** Mingtao Li, Jie Liu, Shunan Chen, Jun Yao, Lei Shi, Hang Chen, Xiaoming Chen

**Affiliations:** 1 Institute of Highland Forest Science, Chinese Academy of Forestry, Kunming 650224, China; mingtaoli2024@163.com (M.L.); chen839071900@163.com (S.C.); eyaojun@hotmail.com (J.Y.); stuchen6481@gmail.com (H.C.); 2 Key Laboratory of Breeding and Utilization of Resource Insects of State Forestry Administration, Institute of Highland Forest Science, Chinese Academy of Forestry, Kunming 650224, China; cafcxm@139.com; 3 College of Forestry, Nanjing Forestry University, Nanjing 210037, China; 4 Institute of Plant Protection, Henan Academy of Agricultural Sciences, Zhengzhou 450002, China; lj014@163.com; 5 Research Center of Resource Insects, Chinese Academy of Forestry, Kunming 650224, China

**Keywords:** *Byasa hedistus*, VOCs, response of visual and olfactory, foraging and courtship

## Abstract

**Simple Summary:**

The butterfly *Byasa hedistus* exhibits strong volatiles but shows no significant differences in color and shape. We analyzed the volatile organic compounds (VOCs) and tested the visual and olfactory behavioral responses of *B. hedistus* during foraging and courtship. The effects of colors and VOCs on the foraging and courtship of *B. hedistus* are discussed. This study enhances our understanding of how male and female butterflies of similar coloration use different signals for foraging and courtship.

**Abstract:**

Color and odor are crucial cues for butterflies during foraging and courtship. While most sexual dimorphic butterflies rely more on vision, our understanding of how butterflies with similar coloration use different signals remains limited. This study investigated the visual and olfactory behavioral responses of the similarly colored butterfly *Byasa hedistus* during foraging and courtship. While visiting artificial flowers of different colors, we found that *B. hedistus* exhibits an innate color preference, with a sequence of preferences for red, purple, and blue. The frequency of flower visits by *B. hedistus* significantly increased when honey water was sprayed on the artificial flowers, but it hardly visited apetalous branches with honey water. This proves that locating nectar sources by odor alone is difficult in the absence of floral color guides. During courtship, males are active while females hardly chase; only two models were observed: males chasing males and males chasing females. The courtship process includes four behaviors: slowing approach, straight chasing, hovering, and spinning. *B. hedistus* cannot distinguish between sexes based on color, as there is no significant difference in color and shape between them. Twenty-three VOCs (>1%) were identified in *B. hedistus*, with 21 shared by both sexes, while ketones are specific to males. These VOCs are principally represented by cineole, β-pinene, and linalool. When cineole was added to butterfly mimics, many butterflies were attracted to them, but the butterflies did not seem to distinguish between males and females. This suggests that cineole may be the feature VOC for identifying conspecific groups. Adding β-pinene and linalool to mimics induced numerous butterflies to chase, hover, spin around, and attempt to mate with them. This suggests that β-pinene and linalool are crucial cues indicating the presence of females.

## 1. Introduction

Survival and reproduction are fundamental themes for species continuity. Butterflies, whether feeding on nectar or fruit juices, store energy for vital activities such as courtship. Most butterflies are brightly colored and slow-flying, making them easily detectable by predators during foraging and courtship. Therefore, to save time and minimize predation risk, butterflies require indicative signals for quickly locating food and mates. The coordination of vision and olfaction plays a crucial role in this process [1,2,3,4]. However, different butterfly species vary in their reliance on vision and olfaction during foraging and courtship [3,4].

Although floral volatiles help attract specific insect populations for foraging [5,6], visual signals transmitted by the corolla (shape, size, and color) may play a more significant role in the foraging process of butterflies [3], especially color traits [7], as butterflies possess more advanced color vision than most insects [8]. For example, in natural environments, the corolla of *Quisqualis indica* (Combretaceae) changes from white to red (accompanied by a reduction in nectar concentration), prompting a shift in pollinators from moths and beetles to butterflies [9]. The similar species exhibits some degree of color constancy when searching for food, independent of light intensity [7]. For example, *Danaus plexippu* (Lepidoptera, Nymphalidae) [10] prefers orange flowers, *Aporia bieti* (Lepidoptera, Pieridae) [11] prefers yellow flowers, and *Papilio xuthus* (Lepidoptera, Papilionidae) [12] prefers blue flowers. Although many butterflies are attracted to different color signals, some find it challenging to forage based solely on visual cues. Species such as *Danaus genutia* (Lepidoptera, Nymphalidae), *Idea leuconoe* (Lepidoptera, Nymphalidae) [3], and *Mycalesis mineus* (Lepidoptera, Nymphalidae) [13] can only accurately locate food in the presence of olfactory signals and exhibit special color preferences. Tang et al. [3] categorized butterfly foraging and courtship behaviors into four types based on their reliance on visual and olfactory senses: (1) vision prioritized over olfaction; (2) olfaction prioritized over vision; (3) olfaction and vision equally important; (4) only olfaction used.

During courtship, butterflies utilize visual information such as wing spot patterns, shapes, and colors to locate mates. While the pattern evolution of *Bicyclus* genus (Lepidoptera, Nymphalidae) well explains the influence of shape on courtship [14,15], in sexually dimorphic butterflies, color traits may be more readily recognized. For example, *Pieris rapae* (Lepidoptera, Pieridae) [16] and *Papilio demoleus* (Lepidoptera, Papilionidae) [2] use differences in ultraviolet reflection on male and female wings, *Heliconius erato* (Lepidoptera, Nymphalidae) uses bright red spots [17], *Hypolimnas bolina* (Lepidoptera, Nymphalidae) uses structural color [18], and *Heliconius cydno* (Lepidoptera, Nymphalidae) uses polarized light [19] for courtship. Even artificially induced color traits can be learned and can lead to courtship preferences in *Bicyclus anynana* (Lepidoptera, Nymphalidae) [20]. Fuller and more vivid colors or patterns indicate the health status of male individuals, making them more appealing to females [21].

In butterflies, color-based visual signals may serve as a long-distance recognition mechanism, while olfactory signals, such as volatile organic chemicals (VOCs), may complement visual patterns to enable precise identification [22]. Many male butterflies possess structures that emit distinctive odors to attract mates, such as the odor-tufted setae at the body margins of *Catopsilia pomona* (Lepidoptera, Pieridae) and the androconia scales at the abdominal ends of *Euploea mulciber* (Lepidoptera, Nymphalidae) [23]. However, these butterflies are also believed to recognize mates visually [24]. Olfaction may still play a subordinate role in most sexually dimorphic butterflies, but in species where males and females have similar coloration, such as *Tirumala limniace* (Lepidoptera, Nymphalidae) and *D. genutia*, olfaction plays a more significant role [4].

*Byasa hedistus* (Lepidoptera: Papilionidae) is characterized by a unique fragrance and is a butterfly of significant ecological and aesthetic value [23]. This unique odor primarily emanates from the androconia scales located in the folds at the rear edge of the males’ hind wings, with significant odor differences between males and females [25]. This paper primarily explores the color recognition, olfactory detection, and selection behaviors of *B. hedistus* during foraging and courtship, discussing the collaborative roles and relative importance of visual and olfactory senses in these processes.

## 2. Materials and Methods

### 2.1. Experiment Location

The experiment was conducted at the Middle Yunnan Plateau Experimental Station of the Chinese Academy of Forestry (39°91′71.28″ N, 116°39′71.28″ E, Lufeng, Yunnan, China). All experiments were performed inside a nylon mesh enclosure (12 × 8 × 6 m) on a sunny day, which provided suitable temperatures (25∼33 °C).

### 2.2. Experimental Material

**Figure 1:**
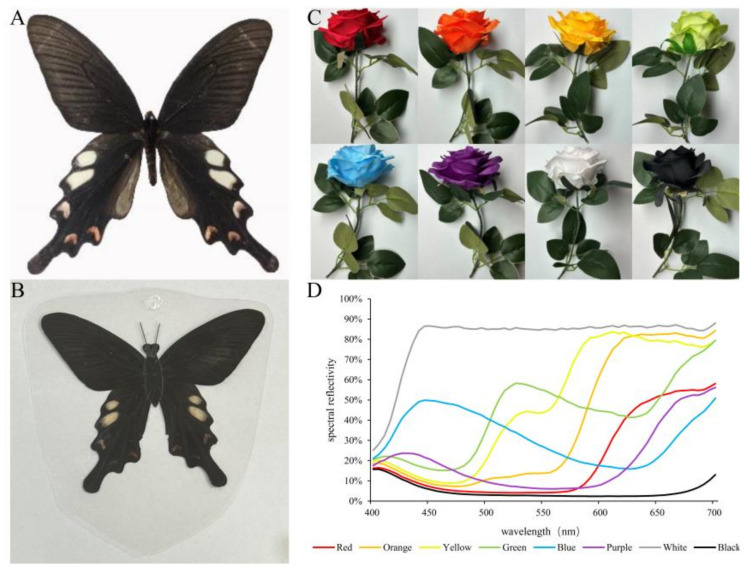
Experimental materials: (**A**) *B. hedistus*; (**B**) butterfly mimics; (**C**) artificial flowers in eight different colors; (**D**) spectral reflectance of the artificial flowers.

Test butterflies: All the butterflies of *Byasa hedistus* (Figure 1A) were collected from the same colony in Lufeng City, Yunnan Province, and artificially bred in a nylon rearing mesh room (24 × 8 × 6 m). Half of the rearing mesh room was covered with black sunshade nets, under which various plants were planted for the butterflies to inhabit. The microclimate was cool and humid. The other half was not covered by sunshade nets while planted with *Aristolochia yunnanensis* (Aristolochiaceae), which is the main host plant of *B. hedistus*. The adults of *B.hedistus* were fed with 10% acacia honey water which sprayed on various colored artificial flowers. Two kinds of nectar plants, *Lantana camara* (Verbenaceae) and *Passiflora caerulea* (Passifloraceae), were planted in the rearing mesh room to supplement the nutrition for the butterflies. The adults of *B.hedistus* lay their eggs on *A. yunnanensis*, and after hatching, the larvae are fed on the leaves of the *A. yunnanensis*.

After the larvae pupating, pupae were hung in the emergence cage (1.5 × 0.5 × 0.8 m). Newly emerged male and female butterflies were placed in different rearing cages (1.5 × 1.5 × 1.5 m). The visiting flower experiment was conducted using butterflies within 2 days of emergence and fed only on clear water (without any color indication related to foraging) before being used in the experiment. The courtship experiment was conducted using butterflies after 3 days of emergence and fed on 10% acacia honey water which sprayed on artificial flowers before being used in the experiment. The emergence cage and rearing cages were placed in the cool rearing mesh room (18–23 °C).

Butterfly mimics: Adult butterflies were prepared by removing the body and appendages. Wings were bonded with black tape, and black plastic antennae were attached. PVC plastic was used to heat-seal the butterfly mimics to completely isolate any odor (Figure 1B).

Artificial flowers: Artificial flowers in eight different colors were made of cotton cloth—red (640 nm), orange (620 nm), yellow (580 nm), green (530 nm), blue (450 nm), purple (430 nm), white, and black—and were selected with similar corolla diameters (8.23 ± 0.11 cm) and depths (2.63 ± 0.05 cm) (Figure 1C). A spectrometer (SOC710VP, Surface Optics Corporation, USA) was used to measure the wavelengths and reflectance of the artificial flowers (Figure 1D).

### 2.3. Observation of the Behavior of Butterflies Visiting Flowers

Before each experiment, newly emerged butterflies, 20 males or 20 females, were selected and placed in the mesh room 24 h in advance to acclimate. The males and females were observed separately. They were fed with clean water only (starvation treatment). Each experiment was repeated three times, using new butterflies for each repetition. 

The artificial flowers, with five flowers of the same color tied together in a bundle, were arranged in a square in the center of the mesh room. The corolla was 1.2 m from the ground, and there was a 2 m distance between the flower bundles. Butterflies visiting flowers were observed daily from 9:30 to 12:00 and 14:00 to 17:30. When a butterfly landed on a flower and extended its proboscis to feed until it curled the proboscis and left, it was recorded as one flower visit.

Artificial flower attraction experiment: During the experiment, 10 mL of clean water was sprayed on the corolla every 30 min, and the positions of the artificial flowers were randomly changed every hour.

Artificial flower + honey water attraction experiment: Honey water was used to represent the VOCs of flowers because it has common VOCs found in flower nectar [26,27], and contains the most important substance benzenoids in guiding the foraging behavior of butterflies [28,29]. The experimental conditions and methods remained essentially unchanged, except that spraying 10 mL of clean water was replaced with spraying 10 mL of 10% fresh acacia honey water [3].

Apetalous branch attraction experiment: The experimental conditions and methods remained essentially unchanged, except the corolla was removed, leaving only the apetalous branches. Then, 10 mL of clean water was sprayed onto the branches every 30 min.

Apetalous branch + honey water attraction experiment: The experimental conditions and methods remained essentially unchanged, except that 10 mL of 10% fresh acacia honey water was sprayed onto the apetalous branches every 30 min.

### 2.4. Observation of the Behavior of Butterfly Courtship

The location, conditions, and observation times were the same as the visiting flower experiment, using unmated butterflies 3 days after emergence. Each test was repeated 3 times, with new butterflies used each time. When a butterfly has multiple behaviors in a single chase, only the final behavior was recorded.

Natural population courtship behavior: Ten male and ten female butterflies were placed in the mesh room, with red dots marked on the white patches of the females’ wings using a marker pen. When an individual adult showed clear approaching behavior toward another, it was recorded as one chase visit. 

Odorless mimic recognition experiment: In the mesh room, 12 mimics (6 female and 6 male) and 6 blanks (only PVC) were vertically hung on three thin white lines, approximately 1.8 m above the ground, with a 1 m distance between mimics. Ten female or ten male butterflies in their courtship period were placed individually in the mesh room, with the positions of the mimics randomly exchanged every two hours, each sex was repeated three times. Each time a butterfly displayed clear approaching behavior (slow down to approach, increase the wing vibration frequency, fly around or contact) toward a mimic, it was counted as one visit. If a butterfly returned to the same mimic within 1 m or 5 s after visiting it, without contacting other mimics, it was only counted as one visit (same below).

Mimic + butterfly grinding slurry recognition experiment: The slurry was made by grinding 10 fresh male or female butterflies, including the body, appendages, and wings, without adding any solvents. The experimental conditions remained unchanged, with 10 male butterflies placed in the mesh room. A 500 μL centrifuge tube was tightly tied to the fine line hanging the mimics, holes were punched in the tube and a cotton ball was placed inside to store volatiles. 

The experiment was divided into two groups: in the first group, 6 male mimics and 3 blanks were filled with 200 μL of male slurry, and 6 female mimics and 3 blanks were filled with 200 μL of female slurry. In the second group, 6 female mimics and 3 blanks were filled with 200 μL of female slurry, and 6 female mimics and 3 blanks were filled with 200 μL of male slurry. Every two hours, the positions of the mimics and blanks were randomly exchanged, and 200 μL of the slurry was replaced using a pipette. 

VOC analysis: Solid-phase microextraction (SPME) was used to extract VOCs from female and male butterflies separately. A 300 mL conical flask was covered with plastic film, and a PDMS/DVB SPME extraction needle (65 μm, Supelco, Bellefonte, PA, USA) was used to extract for 40 min as a blank control. Five female or male butterflies, three days after emergence, were placed in the aforementioned conical flask. After sealing the flask with plastic film, an extraction needle was used to extract the volatile for 40 min. The volatile components were analyzed using a gas chromatography–mass spectrometry (GC–MS) system (TRACE GC ULTRA and ITQ 900 MS, Thermo Fisher Scientific, MA, USA).

Program settings: (1) maintain at 40 °C for 2 min; (2) heat to 120 °C and hold for 2 min (4 °C/min); (3) heat to 230 °C and hold for 5 min (5 °C/min). The inlet temperature was 250 °C, and the carrier gas He was pressurized to 69 kPa [4]. The volatile components were identified using the spectral library of the National Institute of Standards and Technology (NIST, http://webbook.nist.gov/chemistry/, accessed on 6 May 2023). The area normalization method was used to determine the relative content of each volatile compound.

Mimic + VOC recognition experiment: Based on the analysis of the VOCs, the following reagents were selected: cineole (CAS: 470-82-6, 99%, Perfemiker, Shanghai Canspec Scientific & Technology Co., Ltd., Shanghai, China), linalool (CAS: 78-70-6, 99%, Perfemiker), β-pinene (CAS: 127-91-3, 95%, Aladdin, Shanghai Aladdin Biochemical Technology Co., Ltd., Shanghai, China), 4’-methylacetophenone (CAS: 122-00-9, 98%, Aladdin), and 9-fluorenone (CAS: 486-25-9, 99%, Macklin, Shanghai Macklin Biochemical Technology Co., Ltd. Shanghai, China), using n-hexane (CAS: 110-54-3, 99%, Macklin) as the solvent. 

The experiment was divided into four groups: cineole, linalool, β-pinene, and ketone (4’-methylacetophenone + 9-fluorenone). Each group was observed separately.

A total of male butterflies were placed in the mesh room, randomly selecting 3 male mimics, 3 female mimics, and 3 blanks, each filled with 200 μL of 1% volatile reagent. Another set of 3 males, 3 females, and 3 blanks were filled with 200 μL of n-hexane as a control. Every two hours, the positions of the mimics were randomly exchanged, and 200 μL of the volatiles was replenished using a pipette. 

Mimic + different concentration VOC recognition experiment: Based on the data analysis of the VOCs, the experiment was divided into two groups. The first group used a mixture of 0.8% β-pinene + 2.8% linalool to simulate male butterflies, added to 6 male mimics and 3 blanks; a mixture of 2.9% β-pinene + 0.6% linalool was used to simulate female butterflies, added to 6 female mimics and 3 blanks. In the second group, a mixture of 0.8% β-pinene + 2.8% linalool + 0.1% 9-fluorenone + 0.1% 4’-methylacetophenone was used to simulate male butterflies, while the mixture of 2.9% β-pinene + 0.6% linalool was still used to simulate female butterflies. Ten male butterflies were placed in the mesh room for observation. Every two hours, the positions of the mimics were randomly exchanged, and 200 μL of the volatiles was replenished using a pipette.

### 2.5. Color Analysis of Butterfly Specimens and Mimics

A spectrometer was used to measure the wavelengths and reflectance of 3 male specimens, 3 female specimens, 3 male mimics, or 3 female mimics, and the average values were calculated. An ultraviolet meter (WFH-203C, Shanghai Chi Tang Industrial Co., Ltd, Shanghai, China) was used to photograph the ultraviolet reflectance of the wings.

### 2.6. Data Analysis

The data were analyzed using SPSS 25.0 software. A linear mixed model was used to analyze the differences in butterfly color preference. A *t*-test was used to analyze the difference of the number of flower visits between female and male butterflies, the difference between the number of visits before and after spraying honey water on the artificial flowers, the difference between the total number of males chasing males and males chase females, and the differences in courtship behavior (hovering + spinning) during courtship or when chasing butterfly mimics.

## 3. Results

### 3.1. Visual and Olfactory Responses of B. hedistus during Foraging

**Figure 2:**
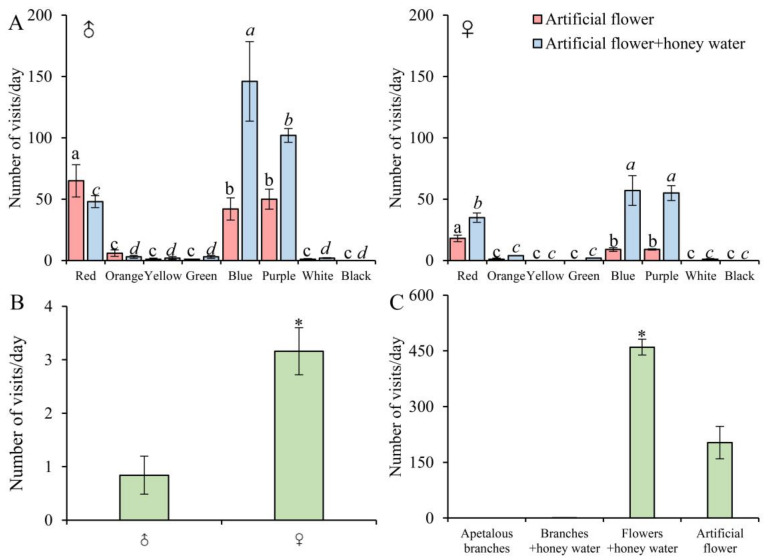
Visual and olfactory responses of *B. hedistus* during foraging. (**A**) The visits of male and female *B. hedistus* to artificial flowers before and after adding honey water; (**B**) fold increase in visits after adding honey water to the artificial flowers; (**C**) total visits of artificial apetalous branches with/without honey water, and total visits of artificial flowers with/without honey water. A linear mixed model was used to analyze the differences in butterfly color preferences. Different letters indicate statistically significant differences (*p* < 0.01). A *t*-test was used to analyze the differences in the number of total flower visits before and after spraying honey water, and the differences of the fold increased times between male and female after adding honey water. * Statistically significant differences (*p* < 0.01).

In the odorless artificial flower experiment (Figure 2A), both male and female butterflies exhibited the strongest preference for red (♂, 39.16% ± 3.39%; ♀, 48.65% ± 3.36%), purple (♂, 30.12% ± 1.27%; ♀, 24.32% ± 2.86%), and blue (♂, 25.30% ± 1.85%; ♀, 24.32% ± 2.63%) (♂, df = 15, F = 88.883, *p* < 0.01; ♀, df = 15, F = 32.523, *p* < 0.01), with few visits to other colors. Males account for 81.71% ± 0.42% of the total flower visits, and females account for 18.29% ± 0.42%. Males were more active, with their flower visit frequency being significantly higher than that of females (df = 4, F = 4.018, *p* < 0.01). After spraying honey water on the artificial flowers, the overall visits increased significantly (average 1.27 times) (df = 4, F = 3.271, *p* < 0.01), with female (3.16 times) were significantly higher than male (0.84 times) (df = 4, F = 2.163, *p* < 0.01) (Figure 2B). This suggests that females are more sensitive to olfactory cues than males under color indication. 

In the experiment of apetalous branches without colors (Figure 2C), *B. hedistus* did not visit green branches (0 times). After adding honey water to the branches (Figure 2C), they still did not visit the branches, except occasionally for resting (average 1 time). This demonstrates that *B. hedistus* finds it difficult to rely solely on odor to accurately locate food sources in the absence of color cues. By comparing the total visits of apetalous branches with honey water (odor only), artificial flowers (color only), and artificial flowers with honey water (color + odor) (Figure 2C), combined with the color preference of *B. hedistus* (Figure 2A), we believe that *B. hedistus* maybe first seek flowers using color cues and then confirm food sources by odors during foraging. This combination of color and odor cues is more advantageous to *B. hedistus* foraging.

### 3.2. Visual and Olfactory Behavior of B. hedistus during Courtship

**Figure 3:**
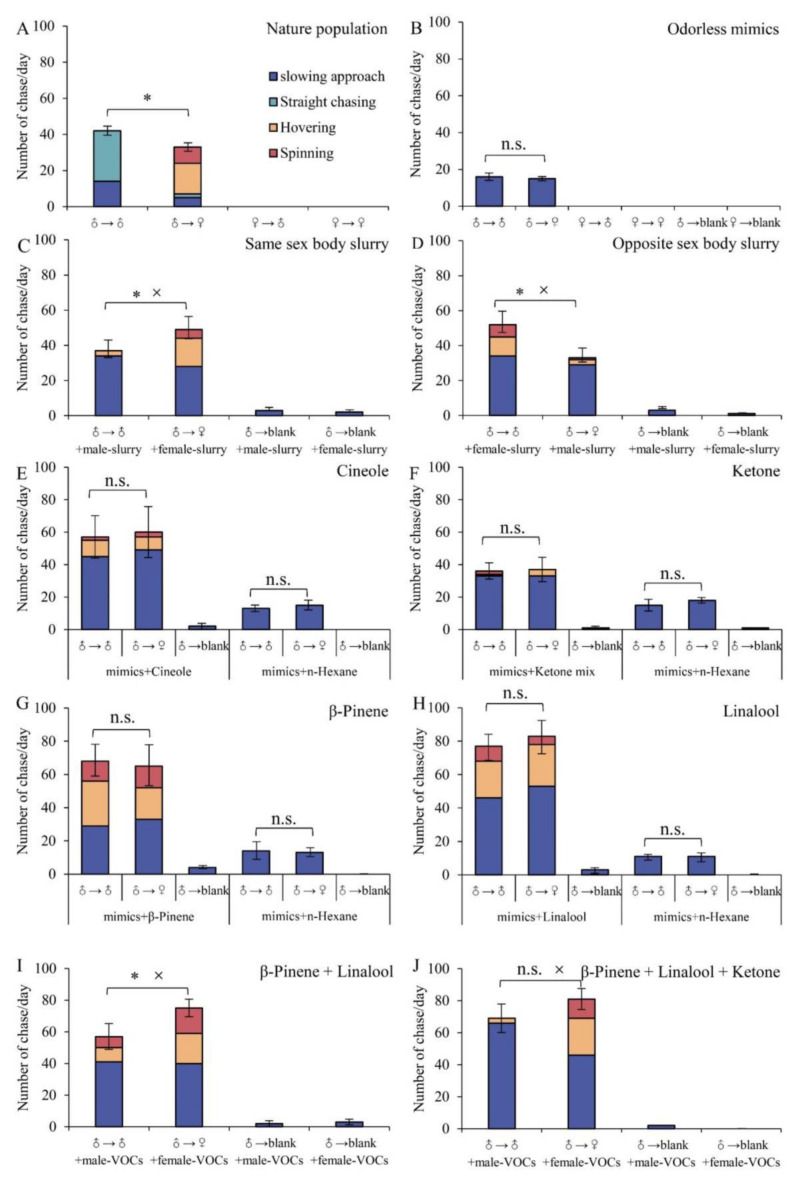
Visual and olfactory recognition during the courtship of *B. hedistus*. (**A**) Natural population courtship; (**B**) visit to odorless mimics; (**C**) visit to mimic + same-sex butterfly grinding slurry, with male mimic + male slurry and female mimic + female slurry; (**D**) visit to mimic + opposite-sex butterfly grinding slurry, with male mimic + female slurry and female mimic + male slurry; (**E**) visit to mimic + 1% cineole; (**F**) visit to mimic + 1% ketone, including 0.5% 9-fluorenone and 0.5% 4’-methylacetophenone; (**G**) visit to mimic + 1% β-pinene; (**H**) visit to mimic + 1% linalool; (**I**) visit to different concentrations of the β-pinene + linalool mixture, using 0.8% β-pinene + 2.8% linalool for male butterflies and 2.9% β-pinene + 0.6% linalool for female butterflies; (**J**) visit to different concentrations of the ketone, β-pinene, and linalool mixture, using 0.8% β-pinene + 2.8% linalool + 0.1% 9-fluorenone + 0.1% 4’-methylacetophenone for male butterflies and 2.9% β-pinene + 0.6% linalool for female butterflies. (**A**–**J**) Analysis using a *t*-test to compare the differences in total visits between males chasing males and males chasing females. Significance is represented by * (*p* < 0.01). (**C**–**J**) Analysis of the differences in courtship behavior (hovering + spinning) between males chasing males and males chasing females. Significance is represented by × (*p* < 0.01). n.s. means no significant difference.

Natural population. During the courtship of *B. hedistus* (Figure 3A), there were two chasing models: males chasing males (56% ± 3.26%) and males chasing females (44% ± 3.26%), which differs from the four chasing models observed in most butterflies (males chasing males, males chasing females, females chasing females, and females chasing males). Males took a very active role, while females were passive. There are four behaviors to distinguish their sex partner: (1) “Slowing approach”, when males find their target and slowly approach to identify if it is female; (2) “straight chasing”, when the target is male or suspected to be male, males dislodge other males or escape themselves through the straight chase; (3) “hovering”, when the target is female or suspected to be female, males hover to distinguish precisely if it is female; and after confirming; (4) “spinning”, when males confirm females, they will spinning around them and looking for a suitable chance to mate. 

When males chase males (Figure 3A), males first use the “slowing approach” behavior. After determining the target as male, one-third fly away after the “slowing approach”, while the other two-thirds engage in rapid “straight chasing” to dislodge other males. When approaching females (Figure 3A), four behaviors occurred: “slowing approach”, “straight chasing”, “hovering”, and “spinning”. The main behavior was “hovering” (51.52% ± 6.37%), where they quickly flapped their wings while holding their bodies upright, and “spinning” (27.27 ± 3.30%), where they slowly flew by twisting their bodies upward to look for a chance to mate.

Odorless butterfly mimics and mimic + butterfly body grinding slurry. The courtship behaviors observed with the mimics are similar to those in natural populations, involving only male chasing male and male chasing female behaviors (Figure 3B), with no visits to blanks. Only the behavior of “slowing approach” was observed when males approached the mimics, and there were no significant differences between male chasing male mimics and male chasing female mimics (df = 4, F = 2.000, *p* = 0.687), indicating that *B. hedistus* can rely on visual cues to find conspecifics but cannot accurately distinguish males or females without odor cues.

In the experiment of mimic + body slurry (Figure 3C,D), after adding body slurry to the mimics, there was a remarkable increase in the number of chases toward the mimics. This could be attributed to the higher concentration of odor in the slurry compared to that emitted by natural butterflies. Males exhibited a preference for female mimics with added female slurry (df = 4, F = 2.909, *p* < 0.01) (Figure 3C), including the behaviors of slowing approach, hovering, and spinning, resulting in an increased frequency of courtship chasing behaviors (42.85% ± 4.02%) (df = 4, F = 4.571, *p* < 0.01). However, only the slowing approach and very few hovering behaviors occurred when males chased male mimics with added male body slurry, while they hardly pursued blank samples despite the addition of body slurry. This indicates that odor serves as a crucial cue for locating potential mates, but without color cues, they can hardly recognize conspecifics. When female body slurry was added to the male mimics and male body slurry was added to the female mimics (Figure 3D), the male chasing behaviors were the opposite. Males prefer to chase male mimics (df = 4, F = 0.174, *p* < 0.01), with hovering and spinning behaviors occurring in the male mimics with female body slurry (34.62% ± 1.85%) (df = 4, F = 4.000, *p* < 0.01). Obviously, inverse body slurry confuses males when identifying both sexes. 

Analysis of VOCs. We analyzed the VOCs of male and female *B. hedistus* and identified 23 volatile substances (>0.1%, Table 1). Twenty-one volatiles were shared between males and females, while two volatiles were unique to males. The most abundant volatiles in both the males and females were cineole (♂ 49.57%, ♀ 51.77%), linalool (♂ 27.61%, ♀ 5.56%), and β-pinene (♂ 8.3%, ♀ 29.37%). Male-specific volatiles included 4’-methylacetophenone (0.14%) and 9-fluorenone (0.1%).

Mimics + VOCs. Four groups of experiments were designed based on the analysis of VOCs, using five volatiles from this butterfly: β-pinene, cineole, linalool, and ketone (4’-methylacetophenone + 9-fluorenone) (Figure 3E–H). The results showed that mimics with added VOCs attracted male butterflies more strongly than the natural population due to their high concentration of VOCs. Blanks (control samples) with added VOCs also induced male chasing, but to a much lesser extent compared to the butterfly mimics (Figure 3E–H), indicating that VOCs play an important role in stimulating male butterflies to identify potential mates. 

Both females and males had a high content of cineole (♂ 49.57%, ♀ 51.77%), which attracted more male chasing but did not allow males to distinguish between females and males (Figure 3E), suggesting that it may be an important compound for recognizing conspecific butterflies. Two types of ketones were identified as specific volatiles of males and hardly induced courtship behaviors in males (Figure 3F), suggesting that they may be key compounds for male butterflies to recognize other males. The β-pinene content in females (29.37%) was higher than that in males (8.3%) (df = 4, F = 3.918, *p* < 0.01), and the linalool content in males (27.61%) was higher than in females (5.56%) (df = 4, F = 5.042, *p* < 0.01). Both compounds induced three behaviors, including slowing approach, hovering, and spinning. The courtship behavior induced by β-pinene (49.23% ± 5.65%) or linalool (36.14% ± 3.76%) was significantly higher than that induced by cineole (18.33% ± 2.29%) (β-pinene, df = 4, F = 1.986, *p* < 0.01; linalool, df = 4, F = 1.178, *p* < 0.01) or ketones (8.82% ± 3.23%) (β-pinene, df = 4, F = 1.585, *p* < 0.01; linalool, df = 4, F = 0.706, *p* < 0.01). This process even occurred in the male mimics (Figure 3G,H), highlighting the critical role of β-pinene and linalool in identifying females in *B. hedistus*. 

We replicated the volatile compounds of β-pinene and linalool found in males and females, adjusting their proportions to simulate the VOCs of females and males. To simulate males, we used a combination of 0.8% β-pinene and 2.8% linalool, while for females, we used 2.9% β-pinene and 0.6% linalool. These synthetic volatiles were then separately added to the male and female mimics in our experiments. The results demonstrated that male butterflies exhibited a preference for the female mimics (df = 4, F = 0.600, *p* < 0.01), eliciting a courtship response of 46.67% ± 7.81% (Figure 3I). In contrast, the male mimics only attracted a courtship response of 28.07% ± 1.28%, significantly lower than female mimics (df = 4, F = 1.370, *p* < 0.01). This indicates that the simulated female volatiles were more effective in attracting males for mating pursuits compared to the simulated male volatiles. 

Furthermore, when we supplemented the simulated male volatiles with specific ketone compounds in males (0.1% 9-fluorenone and 0.1% 4’-methylacetophenone), the behavior of most males (95.65% ± 1.83%) was characterized by a slowing approach and then flying away, which only a few males (4.35% ± 1.83%) hovering around the male mimics (Figure 3J), significantly lower than before adding ketones (Figure 3I) (df = 4, F = 1.455, *p* < 0.01). More behaviors such as slowing approach (56.80% ± 3.42%), hovering (28.40% ± 5.30%), and spinning (6.75% ± 2.01%) occurred in the mimics with simulated female volatiles, indicating that ketones are distinctive volatiles for recognizing males in *B. hedistus*. Furthermore, mixed VOCs are more conducive to courtship recognition in *B. hedistus*.

### 3.3. Color and Reflectance Spectra of Wings

**Figure 4:**
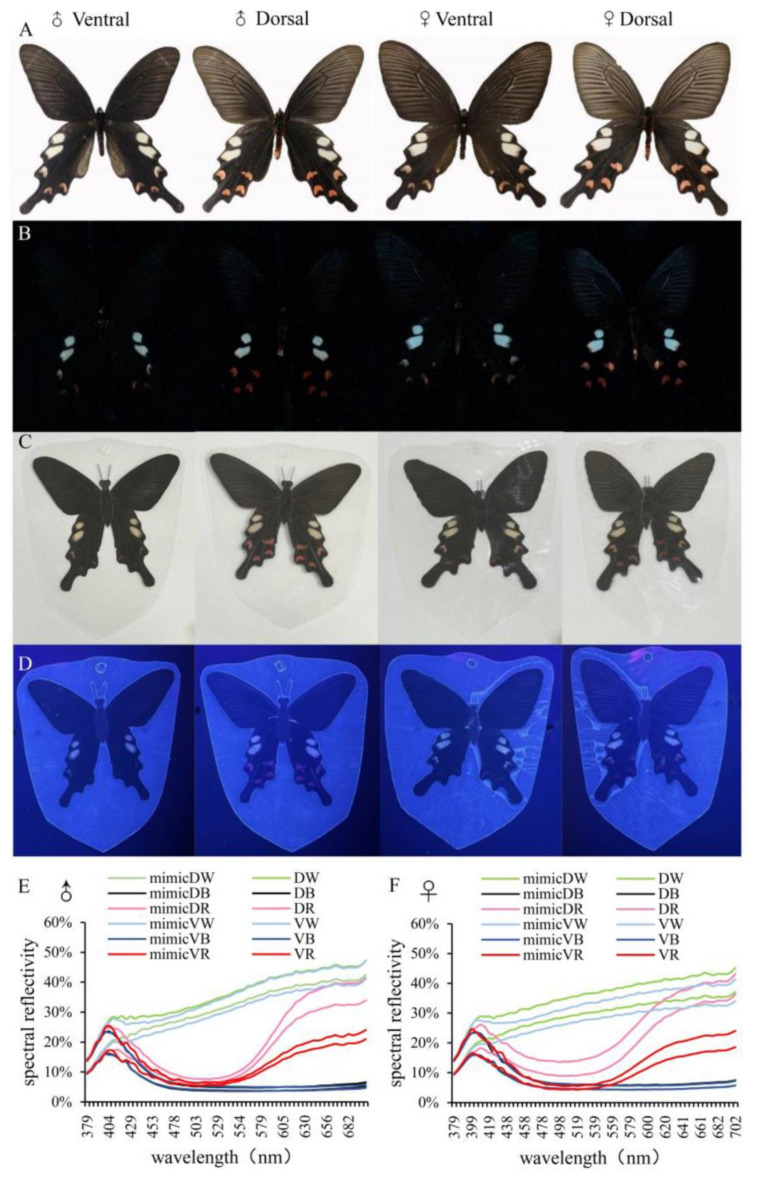
Color and spectral analysis of *B. hedistus*. (**A**) Color of *B. hedistus* under visible light; (**B**) color of *B. hedistus* under UV light; (**C**) color of the mimics under visible light; (**D**) color of the mimics under UV light; (**E**) spectral analysis of male *B. hedistus* and their mimics, where DW means white patches on the dorsal, DB means black patches on the dorsal, DR means red patches on the dorsal, VW means white patches on the ventral, VB means black patches on the ventral, and VR means red patches on the ventral (the same below); (**F**) spectral analysis of female *B. hedistus* and their mimics.

*B. hedistus* primarily have a black base color with white and red patches. The colors are similar between females and males, making them hard to distinguish. Under visible and ultraviolet light, there was almost no visual difference in the patterns and UV reflectance capabilities between males and females (Figure 4A,B). 

Compared to specimens, the mimics showed few visual differences and clearly reflected the colors and patches of male and female butterflies (Figure 4C). Under UV light, the PVC mimics did not interfere with UV reflection; in fact, the mimics appeared clearer (Figure 4D). To further confirm whether PVC encapsulation interferes with color display, a spectrometer was used to analyze the specimens and mimics under UV and visible light spectra (380–700 nm). The spectral curves and main peaks of the three colors between males and females and between specimens and mimics were almost identical, with only slight differences in reflectivity (Figure 4E,F). This suggests that there were no significant differences between the mimics and specimens and little effect on the color vision perception of butterflies. 

## 4. Discussion

Vision and olfaction are essential in the foraging and courtship processes of butterflies. During foraging, both color and odor are important factors influencing butterfly feeding [30,31]. In fact, butterflies prefer flowers with strongly colored petals (e.g., pink, violet, blue, yellow, orange and red) and there are no clear nectar guides [32]. During courtship, butterflies primarily use visual cues to locate conspecifics and identify sex partners based on olfactory signals [33]. However, the visual and olfactory capabilities vary among different butterfly species [3]. Sexually dimorphic butterflies can easily recognize females and males based on differences in wing color and patterns under visible light [17,18] and UV light [2,16], while butterflies with less distinct differences in color and shape mostly rely on olfaction to accurately identify males and females [34].

Our experiments demonstrated that *B. hedistus* possesses developed visual capabilities, showing a preference for red, blue, and purple. *B. hedistus* can easily locate artificial flowers without odors using color cues. More butterflies were attracted to the artificial flowers after odorous honey water was sprayed onto them. However, when honey water was sprayed onto green branches, the butterflies hardly visited them. This indicates that *B. hedistus* finds it difficult to locate food based solely on odor. Many butterfly species of Papilionidae family show strong preference for red, blue and purple flowers, and vision prioritized over olfaction during foraging [1,2,12,24]. However, some species of Nymphalidae, such as *Kallima inachus* and *Danaus genutia*, are thought to rely solely on olfaction for foraging [3,24]. Because relatively high concentrations of floral scent are required to induce the proboscis extension reflex [30,35,36,37], floral scents probably promote foraging behavior only over short ranges for most butterflies [38]. Even in the presence of rewarding yellow flowers, many experienced butterflies preferentially visit unrewarding red flowers [39], indicating that the visual cues were more stable than olfactory cues in most butterflies. *B. hedistus* maybe first seek flowers using color cues and then confirm food sources by odors during foraging. This combination of color and odor cues is more advantageous to *B. hedistus* foraging.

We found that females have a more sensitive olfactory response than males during foraging, which is relatively common in Lepidoptera [1,2,40,41]. This phenomenon may be related to that females rely on odors (including VOCs of leaves and flowers) to locate host plants to complete oviposition [42,43,44].

During courtship, the males of *B. hedistus* chasing females actively, which is the same as most butterfly species [24]. Visual stimuli play a major role in prompting males to flight towards potential mates at long distances [45], which can be used as an initial approach of butterfly for species recognition and mate choice [46,47]. The males of *B. hedistus* approach any target they find through “slowing approach”, including females, other males and even odorless mimics. When males chasing odorless mimics, only “slowing approach” occurred, indicating that *B. hedistus* may first rely on vision to locate a suspected mate at a longer distance during courtship. However, after adding the grinding slurry to mimics, more males were guided to accurately find female mimics, and more “hovering” and “spinning” were happened, suggesting that *B. hedistus* could not identify females by vision alone, but needed to accurately identify females by olfaction at short range. Some “courtship sequences” studies in butterflies with the similar coloration of male and female provide evidence for our finding, such as Pinzar’s study on *Hipparchia statilinus* and *H. Semele* (Lepidoptera, Nymphalidae) [46], and Pliske’s study on *Danaus Plexippus* (Lepidoptera, Nymphalidae) [48]. However, in sexual dimorphism butterflies, such as *Eurema lisa* (Lepidoptera, Pieridae) [49], males can quickly identify females from a distance without approaching their target, and spends significantly less time for seeking mates than the butterflies with the similar coloration of male and female. 

*B. hedistus* cannot easily distinguish between sexes due to the lack of significant discrepancies in their color and shape. However, butterflies of the *Byasa* genus are characterized by a distinctive fragrance [25]. Therefore, we analyzed the VOCs of *B. hedistus* and identified 23 VOCs, including cineole, β-pinene, and linalool. Cineole was the most abundant VOC (♀ 51.77%, ♂ 49.57%) in both sexes of *B. hedistus*, while the main VOC of *Byasa alcinous* is sesquiterpenes [25]. Most species of *Byasa* genus are similar in appearance [23], and differences in the most abundant VOC may relate to interspecific recognition, which can prevent false mating [45,49]. More male chasings were observed when cineole was added to the mimics, but the butterflies could not distinguish between females and males. Due to its high volatility and concentration, cineole may be a characteristic volatile for recognizing conspecifics of *B. hedistus*. However, β-pinene and linalool showed significant differences in concentration between males and females, inducing more courtship behaviors (hovering and spinning), suggesting that β-pinene and linalool may be key substances in courtship. The important substance that stimulates the courtship in *I. leuconoe* and *Cethosia cyane* (Lepidoptera, Nymphalidae) is cycloheptatriene [50] and cedrol [4], respectively, suggesting that the substances that stimulate courtship have strong specificity between different butterfly species. Furthermore, we replicated mixed volatiles based on VOC components of females and males to test the capability of *B. hedistus* to identify both sexes. The results showed that the volatile component of females attracted more males to chase the mimics, and their chasing included all courtship behaviors. This demonstrates that *B. hedistus* distinguishes between sexes based on mixed VOCs rather than relying on a single VOC [51]. There is evidence that male characteristic odors are critical for mating [52]. When ketone was added to the male’s mixed VOCs, most courtship behaviors involved slowing approach (95.65%) with few instances of hovering, proving that ketone is a significant characteristic of males. 

*B. hedistus* has a developed visual system and the ability to locate the nectar of flowers based on color but it cannot identify both sexes by color alone during courtship because there are no significant color differences between sexes. Unlike most other butterflies, the butterflies of the *Byasa* genus are characterized by a distinctive fragrance [25]. The VOCs of *B. hedistus* have strong volatility, and this characteristic compensates for its inability to identify both sexes by vision alone. Mixed VOCs play a crucial role in distinguishing between sexes during courtship. The visual and olfactory characteristics of *B. hedistus* resulted in them visiting flowers and feeding on nectar during foraging using color cues; during courtship, they first found their target butterfly by color and then distinguished between sexes using VOCs.

## Figures and Tables

**Table 1:** VOC analysis of *B. hedistus*.

Retention (min)	Compound	Content (%)	
		♀	♂
11.9	β-Pinene	29.37 ± 2.58	8.3 ± 0.40
13.33	Cineole	51.77 ± 2.55	49.57 ± 4.33
14.01	β-Ocimene	0.48 ± 0.05	3.14 ± 0.71
14.38	Terpineol	0.1 ± 0.01	0.23 ± 0.04
15.49	Terpinolene	2.33 ± 0.47	1.37 ± 0.17
15.96	Linalool	5.56 ± 0.23	27.61 ± 3.47
16.99	Octa-2,4-diene	0.12 ± 0.01	0.41 ± 0.11
17.79	Trimethylpentanediol	1.28 ± 0.11	1.17 ± 0.44
18.32	Terpineol	0.14 ± 0.02	0.09 ± 0.01
18.46	4’-Methylacetophenone	---	0.14 ± 0.01
19.15	2-Butoxyethanol	4.07 ± 0.27	3.95 ± 0.30
20.13	PhenoXyaethanolum	0.04 ± 0.01	0.08 ± 0.02
21.44	Isoquinoline	0.13 ± 0.03	0.18 ± 0.03
22.17	1-Indanone	0.27 ± 0.05	0.3 ± 0.02
23.17	Tridecane	0.1 ± 0.01	0.11 ± 0.01
25.55	3-Methylicosane	0.04 ± 0.01	0.04 ± 0.01
25.73	3-Methylnonadecane	0.06 ± 0.02	0.07 ± 0.01
26.06	(-)-thujopsene	0.27 ± 0.03	0.36 ± 0.08
26.81	β-elemene	0.85 ± 0.17	0.33 ± 0.05
28.29	C15H24	0.73 ± 0.19	0.76 ± 0.33
29.83	β-Caryophyllene	1.83 ± 0.29	1.16 ± 0.13
30.26	9-Fluorenone	---	0.1 ± 0.01
34.28	6-Methylnonadecane	0.33 ± 0.04	0.46 ±0.08

## Data Availability

Data are available on request from the authors.
